# Supplementary material for: Development of a Machine Learning Model to Predict Non-Durable Response to Anti-TNF Therapy in Crohn’s Disease Using Transcriptome Imputed from Genotypes
Source: J Pers Med. 2022 Jun 9;12(6):947. doi: 10.3390/jpm12060947 (PMC9224874; doi:10.3390/jpm12060947)

Figure S1. Performance of the models with various tissue model of PrediXcan, selecting the top significant feature for classifying a non-durable response versus a durable response. The model training by 5-fold cross-validation and feature selection via Least Absolute Shrinkage and Selection Operator and recursive feature elimination was repeated 100 times (see main text for details). The top one feature was turned out to be a gene expression feature for each tissue (Table 2). The training and test performances, given as area under the receiver operating characteristic curve (AUC-ROC, ***upper panel***) and precision-recall curve (AUC-PRC, ***lower panel***), are shown as boxplots. The whole blood model performed the best.

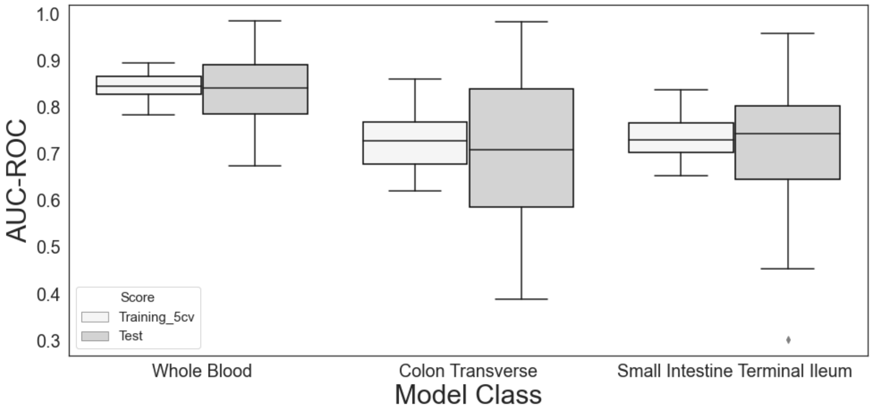

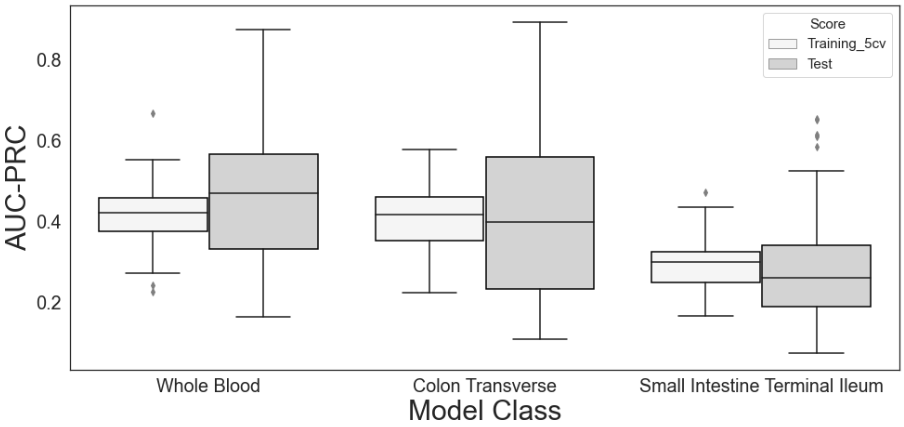

Figure S2. Representative ROC and PRC curves of the test set for various tissue models. For each repetition as in Figure S1, a cutoff was defined where the Youden's index was maximal and the sensitive (recall), specificity, and precision were measured. Among the repetitions, a case whose metrics resemble the mean values was looked for and plotted for ROC (left panel) and PRC (right panel). The dotted lines represent random predictions, which are a diagonal line with slope of 1 for ROC, and a horizontal line with an intercept of the case proportion in the test set for PRC.

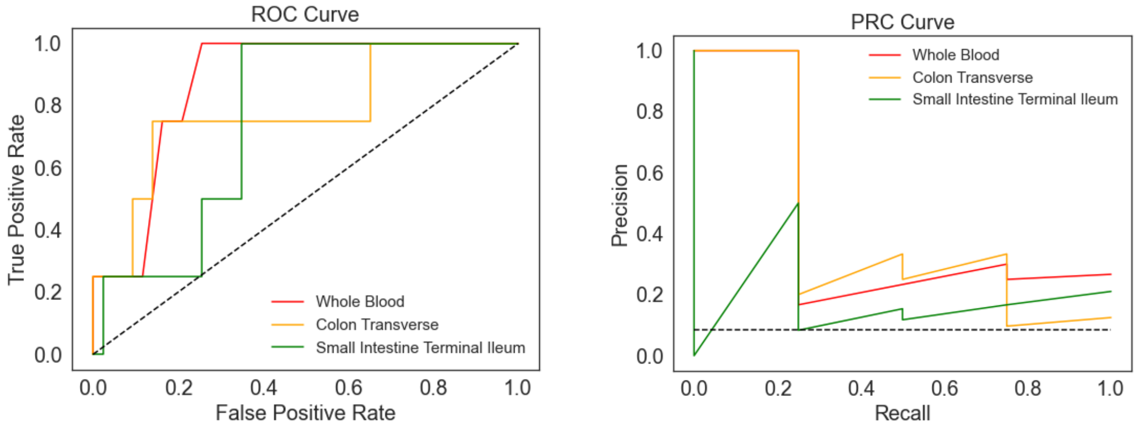

Figure S3. Performance of the models with increasing number of selected features for classifying a non-durable response versus a durable response (Table 3). The model training by 5-fold cross-validation and feature selection via Least Absolute Shrinkage and Selection Operator and recursive feature elimination was repeated 100 times (see main text for details). The top one, two, or three significant features were selected; the clinical features were not among the top features. The training and test performances, given as area under the receiver operating characteristic curve (AUC-ROC, ***upper panel***) and precision-recall curve (AUC-PRC, ***lower panel***), are shown as boxplots. As more features were included, performance improved.

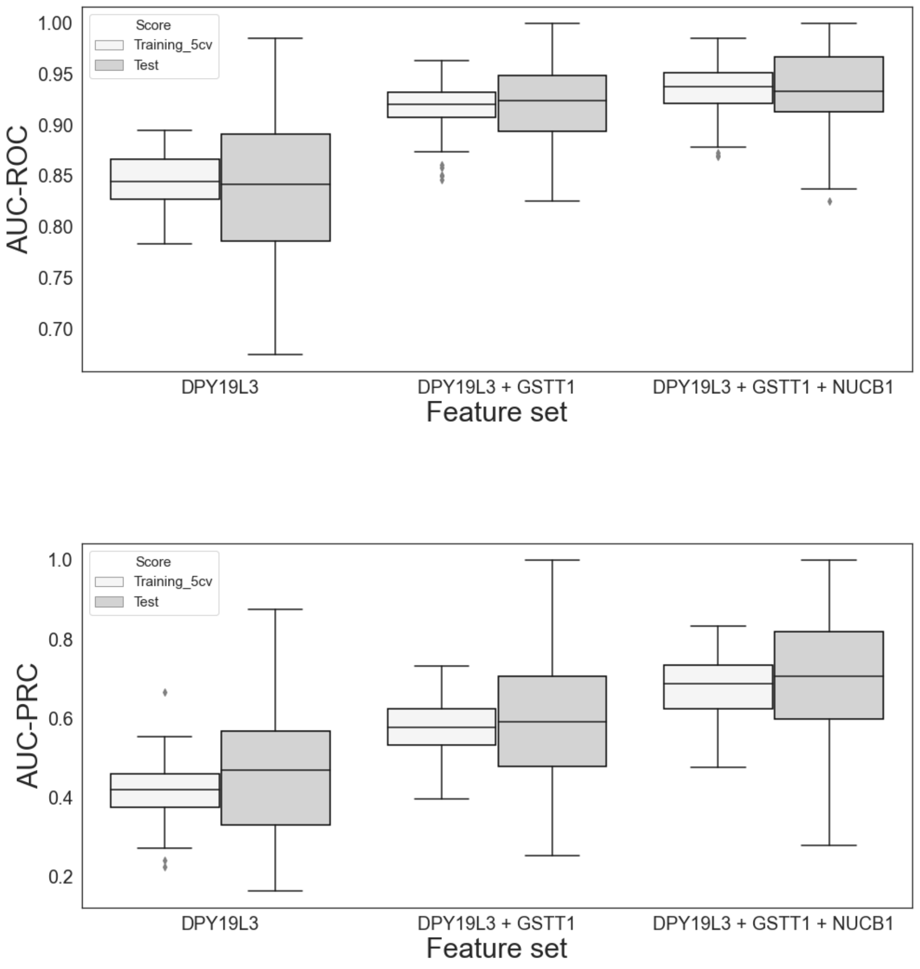

Figure S4. Representative ROC and PRC curves of the test set for increasing number of selected features (Table 3). For each repetition as in Figure S3, a cutoff was defined where the Youden's index was maximal and the sensitive (recall), specificity, and precision were measured. Among the repetitions, a case whose metrics resemble the mean values was looked for and plotted for ROC (left panel) and PRC (right panel). The dotted lines represent random predictions, which are a diagonal line with slope of 1 for ROC, and a horizontal line with an intercept of the case proportion in the test set for PRC.

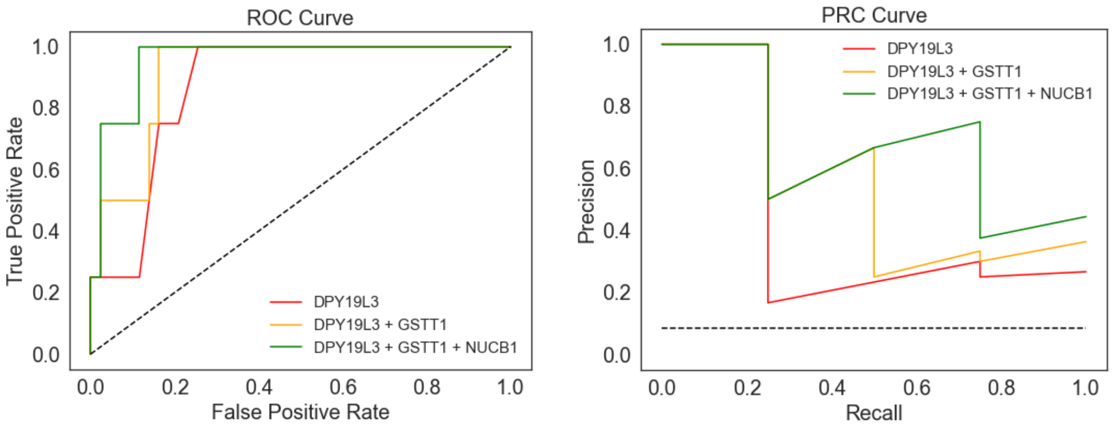

Figure S5. Performance of the models with increasing number of genetic features (Table 3) and fixed contribution of clinical features for classifying a non-durable response versus a durable response. The model training by 5-fold cross-validation and feature selection via Least Absolute Shrinkage and Selection Operator and recursive feature elimination was repeated 100 times (see main text for details). The top one, two, or three significant features as listed in Table 3 were combined with a fixed contribution of clinical features; no feature selection was applied to the clinical features. The training and test performances, given as area under the receiver operating characteristic curve (AUC-ROC, ***upper panel***) and precision-recall curve (AUC-PRC, ***lower panel***), are shown as boxplots. As more features were included, performance improved.

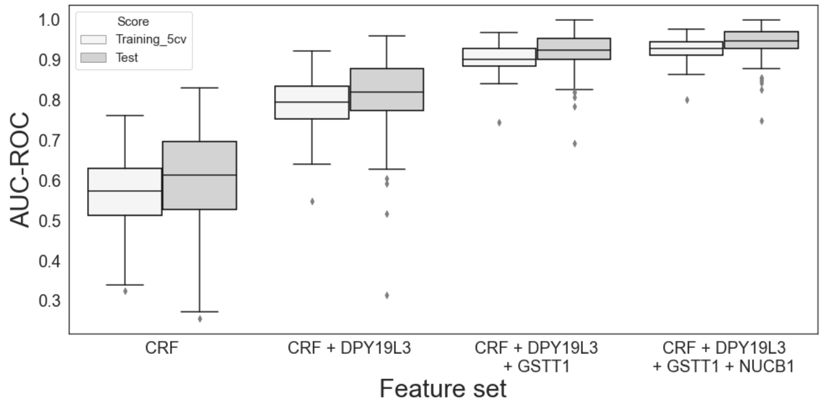

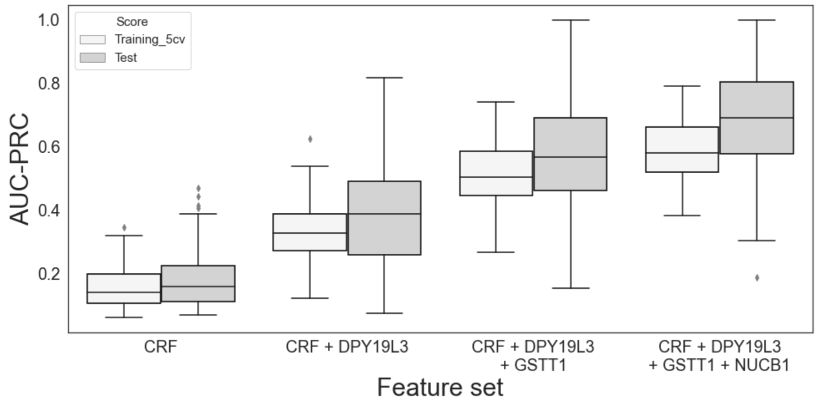

Figure S6. Representative ROC and PRC curves of the test set for the selected genetic features with a fixed contribution of clinical features. For each repetition as in Figure S5, a cutoff was defined where the Youden's index was maximal and the sensitive (recall), specificity, and precision were measured. Among the repetitions, a case whose metrics resemble the mean values was looked for and plotted for ROC (left panel) and PRC (right panel). The dotted lines represent random predictions, which are a diagonal line with slope of 1 for ROC, and a horizontal line with an intercept of the case proportion in the test set for PRC.

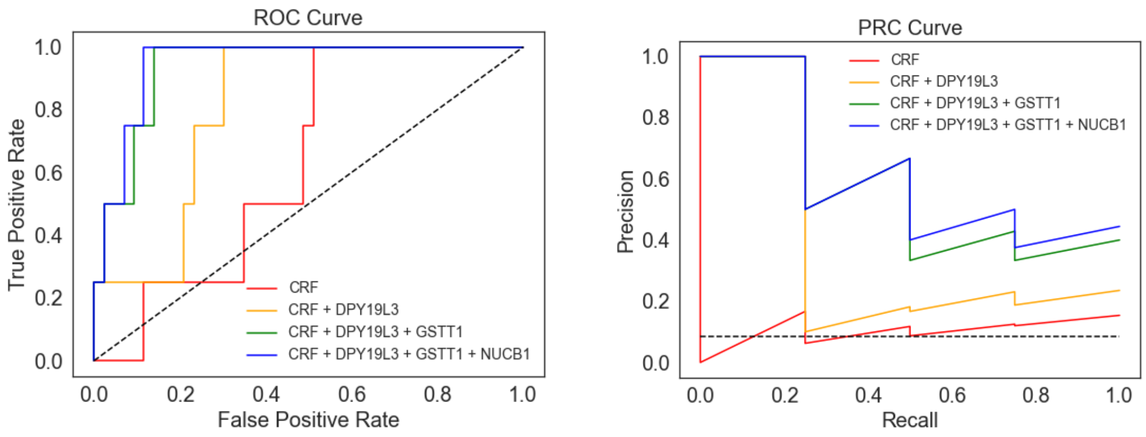

Supplement: Supplementary file 1 [file jpm-12-00947-s001.zip › Supple Figures S1¿CS6 .pdf]
