# Supplementary material for: Development of a Machine Learning Model to Predict Non-Durable Response to Anti-TNF Therapy in Crohn’s Disease Using Transcriptome Imputed from Genotypes
Source: J Pers Med. 2022 Jun 9;12(6):947. doi: 10.3390/jpm12060947 (PMC9224874; doi:10.3390/jpm12060947)
Supplement: Supplementary file 1 [file jpm-12-00947-s001.zip › Supple Tables S1¿CS4 .pdf]

Table S1. Average performance of various tissue models with the top significant feature

Tissue of PrediXcan model	Selected features	Feature selection frequency out of 100 repeats	AUC-ROC (SD)		AUC-PRC (SD)	
			Training 5CV set	Test set	Training 5CV set	Test set
Whole Blood	*DPY19L3*	79	0.845 (0.027)	0.839 (0.070)	0.418 (0.072)	0.460 (0.169)
Colon Transverse	*TXNDC16*	40	0.728 (0.060)	0.711 (0.152)	0.409 (0.081)	0.416 (0.199)
Small Intestine Terminal Ileum	*ENSG00000270127*	14	0.738 (0.045)	0.720 (0.120)	0.291 (0.061)	(0.129)

5-CV, 5-fold cross-validation; AUC-ROC, area under the receiver operating characteristic curve; AUC-PRC, area under the precision-recall curve

Table S2. Average performance of the whole blood models without clinical features

Feature selection frequency out of 100 repeats	Selected features	AUC-ROC (SD)		AUC-PRC (SD)	
		Training 5CV set	Test set	Training 5CV set	Test set
79	*DPY19L3*	0.845 (0.027)	0.839 (0.070)	0.418 (0.072)	0.460 (0.169)
32	*DPY19L3, GSTT1*	0.918 (0.023)	0.919 (0.040)	0.571 (0.078)	0.595 (0.160)
9	*DPY19L3, GSTT1, NUCB1*	0.935 (0.024)	0.935 (0.041)	0.676 (0.080)	0.700 (0.157)

5-CV, 5-fold cross-validation; AUC-ROC, area under the receiver operating characteristic curve; AUC-PRC, area under the precision-recall curve

Table S3. Average performance of the whole blood models with fixed clinical features

No. of features	Features	AUC-ROC (SD)		AUC-PRC (SD)	
		Training 5CV set	Test set	Training 5CV set	Test set
8	*CRF*	0.568 (0.088)	0.603 (0.117)	0.155 (0.062)	0.186 (0.097)
9	*CRF + DPY19L3*	0.788 (0.058)	0.811 (0.102)	0.328 (0.093)	0.390 (0.157)
10	*CRF + DPY19L3, GSTT1*	0.903 (0.034)	0.920 (0.050)	0.513 (0.097)	0.580 (0.170)
11	*CRF + DPY19L3, GSTT1, NUCB1*	0.928 (0.028)	0.943 (0.042)	0.587 (0.093)	0.675 (0.172)

5-CV, 5-fold cross-validation; AUC-ROC, area under the receiver operating characteristic curve; AUC-PRC, area under the precision-recall curve

Table S4. Average sensitivity, specificity, and precision of the whole blood models with the test set

Features		Sensitivity (SD)*	Specificity (SD)*	Precision (SD)*
-CRF	DPY19L3	0.920 (0.128)	0.720 (0.166)	0.306 (0.189)
	DPY19L3, GSTT1	0.998 (0.025)	0.831 (0.079)	0.396 (0.146)
	DPY19L3, GSTT1, NUCB1	0.985 (0.060)	0.846 (0.100)	0.456 (0.223)
+CRF	-	0.855 (0.198)	0.528 (0.196)	0.169 (0.089)
	DPY19L3	0.930 (0.129)	0.712 (0.160)	0.283 (0.147)
	DPY19L3, GSTT1	0.998 (0.025)	0.833 (0.099)	0.418 (0.180)
	DPY19L3, GSTT1, NUCB1	0.990 (0.049)	0.879 (0.081)	0.488 (0.169)

*Model training by 5-fold cross-validation and evaluation with test set was repeated 100 times. For each run, a cutoff dividing NDR vs DR was determined as the point where the Youden's index of ROC was maximal. The sensitivity, specificity, and precision were then measured.
